# Warming has limited effects on plant growth through nutrient release: evidence from sub-Antarctic Marion Island

**DOI:** 10.1093/aob/mcaf154

**Published:** 2025-07-18

**Authors:** Nita C M Pallett, Brad S Ripley, Michelle Greve, Michael D Cramer

**Affiliations:** Department of Biological Sciences, University of Cape Town, Private Bag X1, Rondebosch, Cape Town 7701, South Africa; Department of Botany, Rhodes University, PO Box 94, Makhanda 6140, South Africa; Department of Plant and Soil Sciences, University of Pretoria, Private Bag X20, Hatfield, Pretoria 0083, South Africa; Department of Biological Sciences, University of Cape Town, Private Bag X1, Rondebosch, Cape Town 7701, South Africa

**Keywords:** Soil warming, cold ecosystems, climate change, plant productivity, nutrient release, *Poa annua*, *Polypogon magellanicus*, *Agrostis stolonifera*, *Poa cookii*

## Abstract

**Background and Aims:**

Cold ecosystem plant productivity is nutrient-limited, largely due to temperature-limited soil decomposition rates. Climate warming is predicted to indirectly stimulate productivity by stimulating microbial activity and thus nutrient release. However, these trends are not consistent across cold systems, and the predictions require empirical testing. Here, we investigated whether soil warming on sub-Antarctic Marion Island (49.9°S, 37.8°E) indirectly stimulates grass productivity through increased nutrient release.

**Methods:**

Four grasses (native *Polypogon magellanicus* and *Poa cookii* and alien *Poa annua* and *Agrostis stolonifera*) were subjected to soil warming (ambient +3 °C) in a potted experiment for 5 months. A second experiment with a fertilizer (NPK) treatment tested for nutrient limitation under warming. Additionally, soils with varying organic content were incubated at 5 °C (control), +3 °C and +6 °C for 42 d to determine changes in soil and microbial C, N and P.

**Key Results:**

Warming consistently increased plant growth for only one species (the invasive alien *Poa annua*), but increased leaf N overall. Warming increased soil NH_4_^+^ but NO_3_^−^, organic N and PO_4_^3−^ remained unchanged, and warming had small or non-significant effects on microbial biomass, N and P. In contrast to warming alone, NPK fertilization stimulated growth at least two-fold and increased leaf N, showing nutrient limitation to growth despite soil warming.

**Conclusions:**

It is important to empirically test the assumption of nutrient release with cold-ecosystem warming, and we show that warming-induced nutrient release should not be assumed. Only the ruderal and phenotypically plastic *Poa annua* increased above-ground biomass with soil warming, indicating that nutrient release on Marion Island is limited even with short-term warming (<1 year). Nutrient release with warming is likely not a major driver of vegetation change on Marion Island.

## INTRODUCTION

Plant productivity in cold ecosystems is nutrient-limited due to temperature limitations on soil decomposition rates (Chapin, 1983). These soil decomposition processes are more sensitive to temperature increases than plant productivity due to a higher Q_10_ (change in respiration with a 10 °C temperature increase). This temperature sensitivity increases with decreasing ambient temperatures, i.e. decomposition is highly sensitive to rising temperatures in cold ecosystems (Kirschbaum, 1995). This suggests that warming in cold regions will have a stronger effect on plant growth indirectly, by stimulating soil nutrient release, than directly by stimulating cell division and enzyme activity (Kirschbaum, 1995, 2000). Global climate change increases mean annual and growing season temperatures (IPCC, 2022), and warming in high-latitude ecosystems has been linked to changes in vegetation productivity over the last few decades (Elmendorf *et al*., 2012; Keenan and Riley, 2018). However, the role of nutrient release on vegetation changes such as plant productivity in these high-latitude systems still warrants empirical testing.

There is evidence for responses in plant productivity and nutrition under experimental soil warming. The mineralization of soil organic matter (SOM) by microorganisms releases inorganic nutrients such as N (e.g. NH_4_^+^, which can be nitrified to NO_3_^−^) and P (PO_4_^3−^). Experimental warming may increase microbial mineralization rates and soil inorganic N (iN; the sum of NH_4_^+^ and NO_3_^−^), and this has been linked to increases in plant biomass in the Arctic tundra (Sistla *et al*., 2013). Changes to soil nutrient concentrations also influence plant nutrition. For example, warming-induced increases in soil iN have been linked to increased plant leaf N (%), indicating higher N uptake, in the Arctic (Sistla *et al*., 2013) and in global meta-analyses (Bai *et al*., 2013). The stimulation of microbial activity also increases extracellular enzyme activity, releasing bioavailable organic N (oN, e.g. free amino acids) (Brzostek *et al*., 2012), which can contribute to plant nutrition. For example, warming in the Qinghai–Tibetan plateau increased soil oN concentrations, leading to higher plant oN acquisition (Jiang *et al*., 2018) and altered community composition (Pang *et al*., 2019).

Despite the wealth of literature investigating how warming influences cold ecosystem plant productivity, the extent to which nutrient release occurs and how plants respond warrant further investigation. For example, despite a general trend of increased iN with warming in meta-analyses, some cold, high-latitude sites showed no change or indeed a decrease in soil iN (Rustad *et al*., 2001; Bai *et al*., 2013). Furthermore, warming-stimulated soil decomposition may be a transient effect, decreasing over time (Romero-Olivares *et al*., 2017). However, decreasing stimulatory effects of warming may take years. For example, a meta-analysis showed that the positive response in soil respiration under warming disappeared following *ca.* 7 years of warming (Romero-Olivares *et al*., 2017).

Other confounding variables, such as water availability or plant litter C:N ratio, can limit plant responses to warming (Allison and Treseder, 2008; Beier *et al*., 2008; Steinauer *et al*., 2015). While stimulated microbial activity can lead to nutrient release, microbes may contrastingly immobilize nutrients, rendering them unavailable for plant uptake (Rasmussen *et al*., 2024). Nutrient release with warming may thus not be ubiquitous, even in cold systems. This challenges early predictions and, importantly, also calls into question many studies that simply assume nutrient release because of warming. For example, some studies have implemented fertilization treatments to simulate the effects of long-term warming (Graglia *et al*., 2001; Schmidt *et al*., 2002; Rinnan *et al*., 2007).

While the effects of warming on cold ecosystem productivity have been widely investigated in northern high latitudes, sub-Antarctic terrestrial systems have received less attention despite similarly high rates of climate warming (le Roux and McGeoch, 2008*a*; Nel *et al*., 2023; van der Merwe *et al*., 2024). Sub-Antarctic islands represent useful study systems for such investigations, as they are relatively simple ecological systems with typically simple nutrient cycles: for example, the lack of grazers results in a detritivore-driven nutrient cycle (Smith, 2002). Moreover, they have a thermo-stable climate, without large temperature ranges between winter and summer (le Roux, 2008). Furthermore, the vegetated soils typically have a high organic content (Smith, 1978), suggesting that alleviating any temperature limitations to bioavailable nutrient release would result in strong plant biomass responses.

This study explored how short-term warming on sub-Antarctic Marion Island (MI; 49.9°S, 37.8°E) affects plant productivity through nutrient release. We hypothesized that soil warming would stimulate grass biomass accumulation directly, but also indirectly through increasing soil N and P. Two potted experiments were run, the first with an ambient and ambient +3 °C treatment and the second with an additional fertilizer (NPK) treatment in a full-factorial design, to test whether plants in the warming treatment were nutrient-limited. A third experiment incubated soils from sites of varying nutrient input (thus varying organic matter and microbial biomass), investigating how organic matter, nutrient concentration and microbial biomass responds to +3 °C and, as a treatment to ensure strong warming stimulation, +6 °C warming.

## MATERIALS AND METHODS

### Study site and species

Marion Island is part of the Prince Edward Islands in the sub-Antarctic Indian Ocean. Sub-Antarctic islands such as MI have a hyperoceanic climate characterized by low but stable temperatures, high rainfall and high winds (Selkirk, 2007). On MI, mean annual temperature is 6 °C with a *ca*. 4 °C seasonal range and annual rainfall is 1640 mm (as measured in 2020) (le Roux, 2008; van der Merwe *et al*., 2024). The island experiences gale-force winds on more than 100 d a year (Schulze, 1971). The soils of sub-Antarctic islands are highly organic from the coast up to *ca*. 300 m a.s.l. Vertebrates that breed on the island, such as pinnipeds and seabirds, result in high but localized nutrient deposition which stimulates local plant growth (Smith, 1978). MI is currently experiencing perturbations in annual temperature, with an overall increase of 0.5 °C between 1949 and 2020 (van der Merwe *et al*., 2024). High rates of warming were identified between the 1950s and 1990s, with a 1.2 °C increase (le Roux and McGeoch, 2008*a*). Mean temperatures then decreased between 2000 and 2009 before increasing again until 2020 (van der Merwe *et al*., 2024).

The grasses in the planted warming experiments, *Polypogon magellanicus* (Lam.) Finot (previously *Agrostis magellanica*), *Poa cookii* (Hook.f.) Hook.f., *Agrostis stolonifera* L. and *Poa annua* L., were collected on MI where they co-occur within 100 m of each other on the east coast (46.88°S, 37.86°E). Two of the species, *Polypogon magellanicus* and *Poa cookii*, are native to MI, while the other two, *Poa annua* and *A. stolonifera*, are anthropogenically introduced and thus considered invasive aliens (Greve *et al*., 2017). The two native MI species show more conservative life-history strategies, with (compared to the invasive species) low growth rates and low phenotypic plasticity (Mathakutha *et al*., 2019). The two introduced species are widespread invasives and have been associated with higher plasticity, e.g. showing photosynthetic acclimation to increasing temperatures (Ripley *et al*., 2020).

Soil cores (20 cm long, 8 cm in diameter) were collected from a site (46.8764°S, 37.8603°E) within 300 m from the grasses with a soil corer, and between five and seven grass tillers were planted in each core. Tillers were sampled from one individual grass, and different grass individuals were sampled for each pot, so each pot with 5–7 tillers constituted one replicate. For the duration of each warming experiment, pots were kept outdoors under natural light and rainfall conditions.

For a short-term, high temperature incubation, soil cores (20 cm long, 8 cm in diameter) were collected from five sites. Different vertebrate influence at each of these sites resulted in a gradient of soil organic content, nutrient input and microbial biomass (Supplementary Data Table S1). Vertebrate influence was categorized based on the proximity to vertebrate colonies. Soils were collected in May 2020, and stored at 5 °C in polyethylene zip-lock bags until incubation in November 2020. The site with low vertebrate activity (Table S1, ‘a’) was the same site from which soils were excavated for both planting experiments. Very few soil invertebrates were present in the soil cores (Table S1). These were assumed to represent the current low natural abundances (invertebrate abundance is currently low on the island due to high invertebrate predation by the invasive house mouse *Mus musculus*, McClelland *et al*., 2018).

### Plant growth under soil warming and fertilization

To investigate the effects of warming on grass biomass, the four grass species (*n* = 17–19 per treatment) were planted in pots and either left at ambient temperatures or heated with a heating cable (25 W m^−2^, Thermon BSX 8-2 Heating Cable, Durham, UK) at 15 cm soil depth (Supplementary Data Fig. S1a). Soil temperatures were monitored with five temperature sensors (107 Temperature Probe, Campbell Scientific, USA) in the warmed pots at 5 cm soil depth. Half of the pots were warmed to 3 °C above ambient soil temperatures (the reference being a pot at ambient temperature), with the heating cable output regulated by a measurement and control datalogger (CR1000X, Campbell Scientific). Soil temperatures in ambient and warmed pots were logged for the duration of the experiment (Fig. S1a). This experiment was run during austral winter and is hereafter referred to as ‘W-W’ (Table 1). When this experiment resulted in relatively small responses to warming, a second experiment was designed and implemented the following summer to further investigate whether soil warming alleviated nutrient limitations to plant growth (hereafter referred to as ‘S-WF’; Table 1).

**Table 1.** Summary of the three experiments presented in this study.

Type	Details	Abbreviation	Treatments (levels)	Duration	Variables of interest	*n*
Planted	Winter (potted, outdoors)	W-W	Warming (ambient, +3 °C)	Five months	Grass AGB	17–19
Planted	Summer (potted, outdoors)	S-WF	Warming (ambient, +3 °C) Fertilizer (none, NPK)	Five months	Grass AGB Leaf N, soil iN and PO_4_^3−^	12
Soil incubation	Controlled environment (indoors)	Soil-W	Warming (5 °C, +3 °C, +6 °C)	42 d	Soil TOC, iN, oN, PO_4_^3−^ Microbial C, N, P	10

In the second experiment S-WF, an additional fertilization treatment was added in a full factorial design (*n* = 12). Half of the pots in the warmed and control treatments were fertilized with 0.75 g (28 g N m^−2^, 13 g P m^−2^, 15 g K m^−2^) of slow-release fertilizer (Osmocote Pro 5-6, NPK 19-9-10 + 2 MgO + TE) added to the soil surface. To account for the effects of warming on soil iN and P without plant nutrient uptake, six pots without plants were maintained in the control and heated (without fertilizer) treatments.

The first (W-W) experiment took place from April to September 2019, and the second (S-WF) from November 2019 to April 2020. For both experiments, pots were kept well-watered (untreated rainwater was added if there were more than two consecutive days without rain) to mimic natural conditions, as most habitats on MI where the grasses occur have naturally waterlogged soils. Non-destructive biomass assessments took place every 4 weeks to determine relative growth rates (RGRs), calculated according to Hoffmann and Poorter (2002). In these assessments, tillers, developing and fully expanded leaves (expanded leaves identified by a visible ligule), and inflorescences were counted, and approximate biomass was determined according to a proxy described by Ibrahim (2007). Above-ground plant material was harvested and dried at 70 °C for 48 h to determine plant biomass. For S-WF, this plant material was then ground and analysed for total N content.

### Soil and microbial C, N and P under warming

The effects of warming on soil nutrient concentrations were tested in the S-WF experiment and the short-term soil incubation (hereafter referred to as ‘Soil-W’, as in Table 1). At the end of the S-WF experiment a soil core (*ca*. 20 g) was collected from each pot, sealed in a polyethylene bag and frozen at −20 °C until analyses. For soil analyses, samples were thawed, extracted with 0.5 m K_2_SO_4_ in a 1:5 w/v (soil wet weight/solution volume) ratio through thorough stirring and then 1 h of shaking on an oscillating shaker, and filtered through a 0.45-µm Whatman filter membrane before analyses for soil iN and P (details of analyses below).

The Soil-W incubation experiment tested how warming (to +3 and +6 °C) affected soil (i.e. plant-available) and microbial (i.e. immobilized and not plant-available) C, N and P. Soil cores were collected from five sites (Supplementary Data Table S1) with varying vertebrate influence and thus nutrient deposition, organic matter and microbial activity. Each core was halved; one half was kept as control in a temperature-controlled room (5 °C) and the other was warmed by either +3 or +6 °C relative to the controls using the same heating cable, regulator and data logger setup as the planted experiment (*n* = 10 control-heated pairs per heating treatment per site). After 42 d of incubation, each replicate was separated into sections, one for gravimetric soil water content (SWC; difference in mass between wet and dry soil cores), and the other for determining iN, P (PO_4_^3−^), total organic C (TOC) and total dissolved N (TDN; the sum of oN and iN). Soil moisture levels were maintained by watering with deionized water every 3 days. Soil nutrient and microbial parameters were regressed against SWC to determine whether SWC influenced the results, as there is evidence that excessive soil water confounds warming stimulation of microbial mineralization (Beier *et al*., 2008).

Microbial C, N and P were measured with the chloroform fumigation-extraction technique. In this method, chloroform lyses microbial cells, releasing microbial C (a proxy for biomass), N and P (Vance *et al*., 1987). Fresh soil was fumigated with ethanol-free chloroform in a vacuum desiccator by boiling and venting the desiccator twice before a final boiling, after which the desiccator was left for 24 h before samples were removed (Vance *et al*., 1987). Soils were then immediately extracted with 0.5 m K_2_SO_4_ at a 1:5 w/v ratio (Beck *et al*., 1997), through thorough stirring and then shaking on an oscillating shaker for 1 h. Following K_2_SO_4_ extraction, extracts were centrifuged (Hermle, Germany) at 671 *g* for 15 min, and the supernatant filtered through a 0.45-µm Whatman filter membrane and then stored at −20 °C until analyses for TOC, TDN and PO_4_^3−^. A separate sample of each replicate was extracted without fumigation, and microbial C, N and P were determined as the difference between fumigated and unfumigated samples. The extractability of microbial C, N and P was not accounted for, and therefore the data represent an index of the variables that are comparable between treatments, not absolute amounts.

### Soil inorganic N and P and organic N and C measurements

Soil iN and PO_4_^3−^ from both the S-WF and Soil-W experiments were determined using colorimetric assays on the 0.5 m K_2_SO_4_ extracts. NO_3_^−^ was measured based on the method described by Schnetger and Lehners (2014), where VCl_3_ reduces NO_3_^−^ to NO_2_^−^, which is then captured by Griess reagents (*N*-1-naphthlyethylenediamine dihydrochloride and sulphanilamide). No correction for NO_2_^−^ was made due to negligible concentrations, as determined with Griess reagents. NH_4_^+^ was measured using the indophenol blue method using Berthelot reagents (phenol, nitroprusside, sodium citrate and sodium hypochlorite) (Dorich and Nelson, 1983). PO_4_^3−^ was measured based on the ammonium molybdate method described by Strickland and Parsons (1972), where the reagents ammonium molybdate, ascorbic acid, sulphuric acid and potassium antimonyl-tartrate form a phosphomolybdate complex. Colorimetric absorbance measurements were made with a spectrophotometer (ThermoSpectonic, Helios Epsilon model, Thermo Scientific, USA) (planted experiment) or using a multiplate reader spectrophotometer (incubation experiment) (Multiskan Spectrum, Thermo Electron Corporation, Finland), at 540, 630 and 885 nm for NO_3_^−^, NH_4_^+^ and PO_4_^3−^, respectively.

Soil TOC was measured using a TOC Torch Combustion Analyser (Teledyne Tekmar, USA) where, after an initial sparging with phosphoric acid to eliminate inorganic C, the oxidation of carbon material produced CO_2_, which was measured using non-dispersive infrared detection. Samples were measured against a 100 ppm potassium phthalate (KHP; C_8_H_5_KO_4_) standard made in 0.5 m K_2_SO_4_.

Soil TDN was measured using a protocol based on Yu *et al*. (1994) and Hagedorn and Schleppi (2000), where samples are treated with a persulfate and NaOH reagent and then autoclaved for 1 h, resulting in persulfate oxidation of all TDN compounds to NO_3_^−^. NO_3_^−^ was then measured colorimetrically according to Schnetger and Lehners (2014), as above. Soil oN was calculated as the difference between TDN and iN. The use of 1 mm urea standards resulted in urea oxidation to NO_3_^−^ with 98.7 % accuracy, and the 1.3 % discrepancy was accounted for.

All analyses used standards prepared in 0.5 m K_2_SO_4_. All soil C, N and P data are presented per g (dry weight). Dry weights were recorded for every core in the Soil-W experiment, and for all cores without plants in the S-WF experiment.

### Leaf N analyses

Leaves from the S-WF experiment were analysed to determine how warming and fertilization influenced leaf N. Samples were ground to a fine powder using a ball mill (MM200, Retsch, Germany), and weighed (2 mg) into tin capsules. These were combusted in a Flash 2000 organic elemental analyser (Thermo Scientific, Germany), and gasses were passed into an isotope ratio mass spectrometer (DELTA V Plus IRMS) via a ConFlo IV gas control unit and calibrated according to in-house standards. Total N content (g) was determined on total above-ground biomass. This was determined using the Flash EA 1112 Series (Thermo Scientific, Germany), where 5 mg of finely ground plant biomass was combusted in a high-temperature reactor. The resultant gases were measured by chromatography after separation by retention rates in a chromatograph column, and results calibrated according to in-house standards.

### Statistical analyses

All statistical analyses were performed using R statistical software, v.4.2.2 (R Core Team, 2022). Data from the W-W and S-WF experiments were subjected to analysis of variance (ANOVA), testing interacting effects between the treatments (warming or warming and fertilizer) and species on plant RGR, total biomass, and (in the second experiment) leaf N, total N (g), soil iN and P.

From the Soil-W experiment, the effect of warming and site (of soil core collection) was tested on soil TOC. Thereafter, the effect of temperature on the relationship between soil TOC and soil iN, oN, P, and microbial C, N and P was tested with linear mixed effects models (LMEMs) with core replicate as a random effect to account for the paired experimental design. However, more than half of the models resulted in a singular fit with zero variance for the random effect (core replicate). For these models, the random effect was removed, and a simple linear model was used. Significant differences (*P*-values) and *F*-values from the linear models were determined with an ANOVA. The effect of warming treatments on SWC, and SWC and warming treatments on soil nutrient and microbial parameters were investigated with LMEMs, with site and core replicate as random effects.

All ANOVAs were performed in the ‘car’ package (Fox and Weisberg, 2019), and LMEMs with the ‘lme4’ package (Bates *et al*., 2015). Where there was evidence for a significant effect (*P* < 0.05), post-hoc pairwise comparisons were determined in the ‘emmeans’ package (Lenth, 2023), which calculates Tukey honest significant differences. In the instances where interaction effects were not significant, they were removed and analysed as additive fixed effects. All residuals were checked, and where they did not conform, response variables were log-transformed (for all plant and soil nutrient data) or square-root-transformed (for microbial C and P relative to SWC data) to meet normality and homoscedasticity assumptions.

## RESULTS

### Plant growth under soil warming and fertilisation

In both planted experiments, the warmed soil cores were *ca*. 3°C above ambient soil temperature (Supplementary Data Fig. S1a, b). In the W-W experiment, ambient soil temperature was 4.1 ± 3.0 °C (mean ± SE) and warmed pots 7.0 ± 1.4 °C, and in the S-WF experiment (warming and fertilizer) 9.0 ± 4.5 °C (ambient) and 11.7 ± 1.8 °C (warmed). These temperatures fall in the concurrently measured range of field soil temperatures on MI from five sites (November 2019–April 2020) with a mean 7.6 ± 1.2 °C and range 2.5–25.0 °C (Data Table S2; J. Schoombie and P. C. le Roux, pers. comm.).

**Fig. 1.**
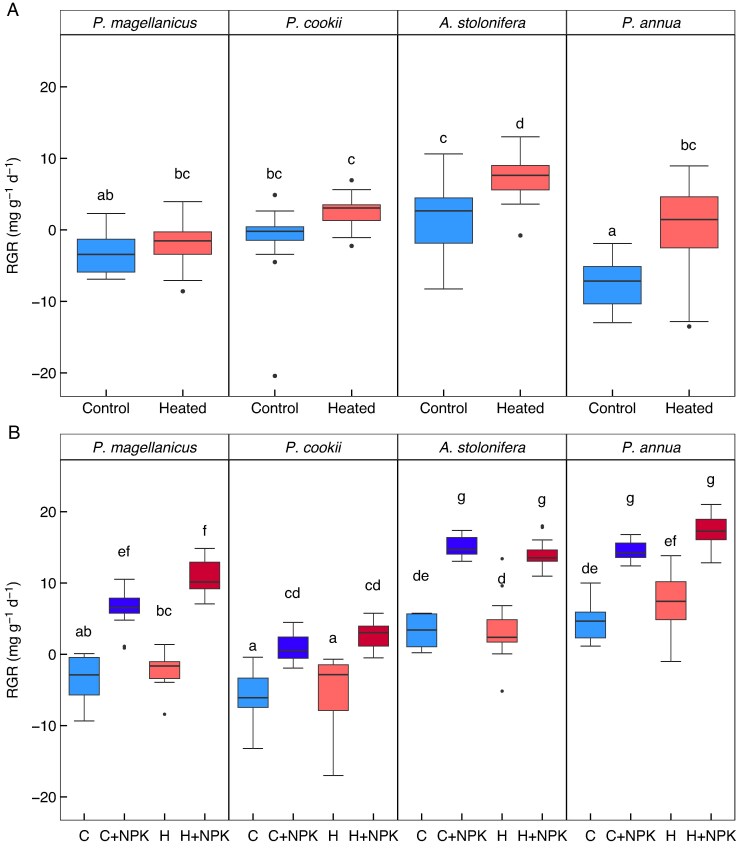
Plant relative growth rate (RGR) for the W-W (A) and S-WF (B) experiments, which had warming treatments, and warming and NPK fertilization treatments, respectively. (A) RGR for W-W, where there was evidence for a significant increase in RGR with warming only for *A. stolonifera* and *Poa annua*. (B) RGR for S-WF. Here, ‘C’ is the control (no warming, no fertilization), ‘C + NPK’ is no warming with fertilization, ‘H’ is warming with no fertilization, and ‘H + NPK’ is warming and fertilization. Despite an ANOVA indicating a significant interaction between species and warming, the post-hoc tests do not show any significantly higher RGR with warming. However, there was a strong effect of NPK fertilization, which increased RGR for all species. Significant differences between treatments and species are denoted by the absence of common letters, as determined by a Tukey HSD post-hoc test at the α = 0.05 significance level.

**Fig. 2.**
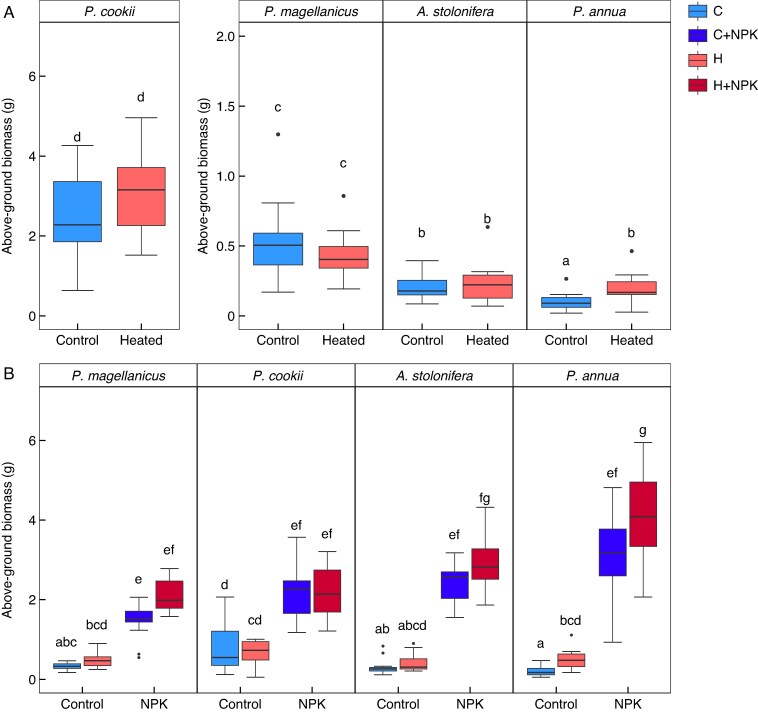
Plant above-ground biomass following the W-W (A) and S-WF (B) experiment, which had warming treatments and warming and NPK fertilization treatments, respectively. (A) W-W above-ground biomass, where there was evidence for a significant increase in above-ground biomass with warming only for *Poa annua*. Note the different *y*-axis scales, due to the higher biomass for *Poa cookii*. (B) S-WF above-ground biomass. As with the W-W experiment, only *Poa annua* had significantly higher biomass with warming. There was a strong increase in above-ground biomass with NPK fertilization, which was significantly higher than the control and warming (no fertilizer) treatments for all species. Significant differences between treatments and species are denoted by the absence of common letters, as determined by a Tukey HSD post-hoc test at the α = 0.05 significance level.

There was a significant interaction between the influence of warming and species on plant RGR and above-ground biomass, with species-specific responses to the higher soil temperatures for both the W-W and S-WF experiments. In the W-W experiment, warming significantly increased RGR for *A. stolonifera* and *Poa annua*, but not for *Polypogon magellanicus* or *Poa cookii* (*F*_3,133_ = 3.51, *P* = 0.017; Fig. 1A). In the S-WF experiment, there was a significant warming and species interaction, but the post-hoc test did not show any species to have significantly higher biomass in the control (no fertilizer) relative to warming (no fertilizer) treatments (*F*_3,180_ = 2.96, *P* = 0.034; Fig. 1B). RGR was negative for many treatments in the W-W experiment, and any positive RGR was relatively low (e.g. *A. stolonifera* control RGR was 1.74 ± 1.17 mg g^−1^ d ^−1^ and *Poa annua* warmed RGR was 0.59 ± 1.52 mg g^−1^ d ^−1^). In the S-WF experiment, RGR was negative for *Polypogon magellanicus* and *Poa cookii* in both the control and the warming treatments, but higher than the W-W experiment for the other two species (e.g. *A. stolonifera* control RGR was 3.31 ± 0.63 mg g^−1^ d ^−1^ and *Poa annua* was 4.76 ± 0.79 mg g^−1^ d ^−1^). Following the experimental harvests, the dried above-ground biomass also resulted in a significant interaction between warming and species (W-W *F*_3,133_ = 3.87, *P* = 0.011; S-WF *F*_3,180_ = 4.63, *P* = 0.004; Fig. 2A, B). Here, only *Poa annua* showed a significant increase in biomass, in both of the experimental harvests.

In the S-WF experiment, NPK fertilization had a substantially stronger effect on grass RGR and final above-ground biomass than warming. Here, there was no evidence for a significant interaction between species, warming and NPK fertilization. However, there was evidence for species-specific responses to fertilization, with a significant NPK–species interaction for RGR (*F*_3,180_ = 4.03, *P* = 0.008; Fig. 1B) and above-ground biomass (*F*_3,180_ = 13.81, *P* < .0001; Fig. 2B). RGR was positive for all species under NPK fertilization, and significantly higher than the unfertilized treatments (control and warmed). For example, compared to the RGR without fertilization, *A. stolonifera* had a higher RGR by 4.56-fold (control and fertilization, 15.09 ± 0.45 mg g^−1^ d ^−1^) and *Poa annua* by 3.04-fold (control and fertilization 14.46 ± 0.41 mg g^−1^ d ^−1^). RGR with fertilizer was significantly higher for both *A. stolonifera* and *Poa annua* compared to the two native species, *Polypogon magellanicus* and *Poa cookii* (Fig. 1B). The dried biomass at harvest showed that all species had significantly higher biomass following NPK fertilization: overall, the increase in biomass with fertilization was 4.49-fold (Fig. 2B). Furthermore, the post-hoc test showed that *Poa annua* had significantly higher biomass with warming and NPK compared to those at ambient temperature with NPK.

**Fig. 3.**
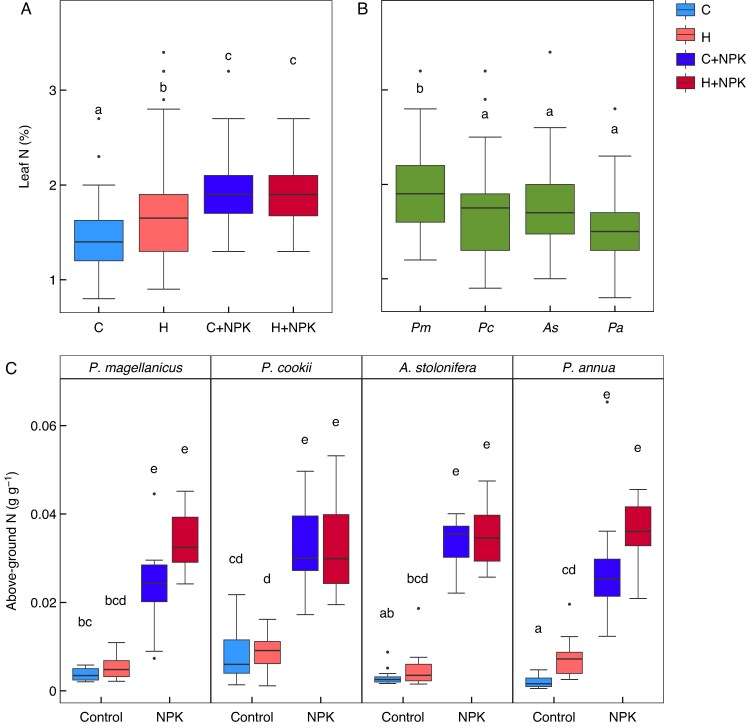
Leaf N (%) and above-ground N (g g^−1^) content analysed for S-WF. (A) Overall leaf N for the warming and NPK treatments. Warming significantly increased leaf N compared to the control in the unfertilized treatment, but NPK fertilization resulted in the highest leaf N. (B) Leaf N for each species, where ‘*Pm*’ is *Polypogon magellanicus*, ‘*Pc*’ is *Poa cookii*, ‘*As*’ is *A. stolonifera* and ‘*Pa*’ is *Poa annua*. Leaf N differed between the species, with significantly higher N (%) for *Polypogon magellanicus*. (C) Total above-ground N content for each species, where there was a significant interaction between species, warming and NPK fertilization. Significant differences between treatments and species are denoted by the absence of common letters, as determined by a Tukey HSD post-hoc test at the α = 0.05 significance level.

The above-ground biomass from the S-WF experiment was analysed for leaf N (%). Here, there was evidence for significant differences between the species, with *Polypogon magellanicus* having significantly higher leaf N% than the other three species (*F*_3,185_ = 9.77, *P* < 0.0001; Fig. 3B). This was unaffected by the treatments. There was a significant interaction between warming and NPK fertilization treatments (*F*_1,185_ = 6.68, *P* = 0.011), where warming increased leaf N in the unfertilized treatments but did not affect the fertilized treatments (Fig. 3A). NPK fertilization resulted in higher leaf N than the unfertilized treatments (Fig. 3A). Total above-ground N content (g g^1^) showed a significant interaction between species, warming and NPK fertilization (*F*_3,176_ = 3.58, *P* = 0.015; Fig. 3C). NPK fertilization significantly increased above-ground N relative to the unfertilized treatments. Warming increased above-ground N relative to the control (no NPK fertilization) for *Poa annua*.

### Soil and microbial C, N and P under warming

**Fig. 4.**
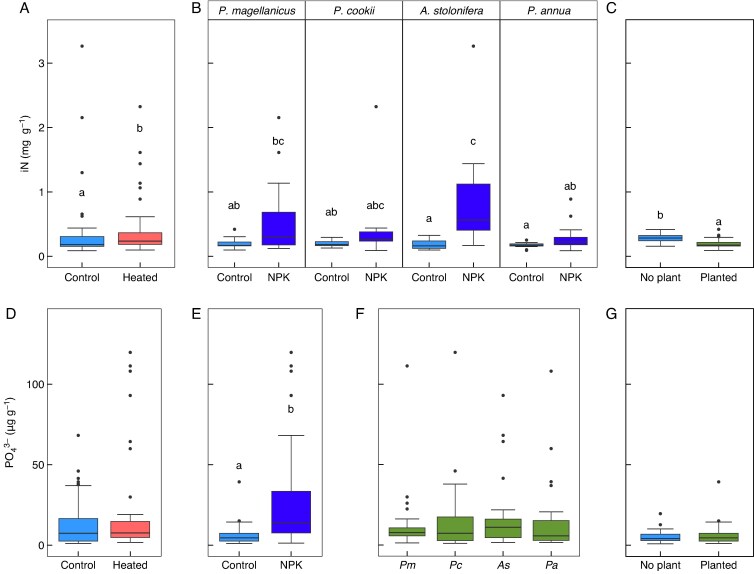
Soil nutrients (iN and PO_4_^3−^) from the S-WF experiment. (A) Soil iN concentrations between the control and heated pots, where iN increased with heating, and (B) with NPK fertilization, where NPK fertilization increased soil iN for pots with *A. stolonifera*. (C) Difference in soil iN between planted and unplanted pots, showing higher iN in unplanted plots. (D) Soil PO_4_^3−^, where there was no significant difference in PO_4_^3−^ concentration between warming treatments. (E) PO_4_^3−^ increased significantly with NPK fertilization, but (F) there was no difference in soil PO_4_^3−^ between species or (G) between planted and unplanted pots. One outlier (NPK treatment, PO_4_^3−^ > 250 µg g^−1^) was removed. Significant differences between treatments and species are denoted by the absence of common letters, as determined by a Tukey HSD post-hoc test at the α = 0.05 significance level.

Soil iN concentrations increased significantly with warming (*F*_1,87_ = 4.10, *P* = 0.046; Fig. 4A) in the S-WF experiment. There was a significant interaction between species and NPK fertilization (*F*_3,_  _87_ = 3.21, *P* = 0.027), where NPK fertilization only significantly increased soil iN for *A. stolonifera* (Fig. 4B). When compared to the pots without plants, the planted pots had significantly lower soil iN (*F*_1,57_ = 15.67, *P* = 0.0002; Fig. 4C). Separate analyses of NH_4_^+^ and NO_3_^−^ showed that the increase with warming was due to a significant increase in NH_4_^+^ but there was no change in NO_3_^−^ (Supplementary Data Fig. S2). Warming had no effect on soil PO_4_^3−^ concentrations, and there was no difference in soil PO_4_^3−^ between species or planted and unplanted pots (Fig. 4D, F, G). NPK fertilization significantly increased soil PO_4_^3−^ (*F*_1,90_ = 39.70, *P* < 0.0001; Fig. 4E).

In the Soil-W experiment, control and the two warming treatments (+3 and +6 °C) resulted in soil core temperatures of 5.10 ± 0.16, 8.03 ± 0.16 and 11.10 ± 0.54 °C, respectively (Supplementary Data Fig. S1C). Soil TOC decreased with warming (Table 2), and there were substantial differences between soil TOC and site (Table 2). Soil iN increased but PO_4_^3−^ decreased with TOC; however, warming did not affect either (Table 2). Soil oN and TOC showed no relationship, and no evidence of a warming effect (Table 2). Microbial C increased with soil TOC, but this relationship did not change with warming (Table 2). There was no relationship between microbial N and soil TOC, and no evidence for a warming effect (Table 2). By contrast, microbial P decreased with soil TOC at +6 °C warming (estimate = −0.13, *t* = −3.18, *P* = 0.0017) but was unaffected at +3 °C warming, as shown by a significant interaction between TOC and warming (Table 2).

**Table 2.** Results from models investigating how soil and microbial nutrients from soils with varying TOC were affected by warming treatments (Soil-W experiment). Non-significant fixed effects are shown as ‘n.s.’, and respective *P*-values are provided for significant fixed effects.

Response variable	Fixed effects	*F*-value (d.f.)	*P*-value
TOC	Warming	3.45 (2,146)	0.034
	Site	69.88 (4,174)	<0.0001
iN	TOC	7.97 (1,193)	0.005
	Warming	1.63 (2,127)	n.s.
oN	TOC	2.58 (1,196)	n.s.
	Warming	1.28 (2,196)	n.s.
PO_4_^3−^	TOC	18.11 (1,196)	0.001
	Warming	2.51 (2,196)	n.s.
Microbial C	TOC	17.79 (1,195)	<0.0001
	Warming	0.06 (2,195)	n.s.
Microbial N	TOC	1.84 (1,186)	n.s.
	Warming	0.62 (2,186)	n.s.
Microbial P	TOC × Warming	4.26 (2,188)	0.015

Soil water content was probably not a confounding factor for warming-induced nutrient release in this experiment: SWC had a negative relationship with only one of the nutrients investigated (NO_3_^−^), and in this case, warming resulted in a less negative relationship between SWC and NO_3_^−^ than the control. For the other soil variables analysed, SWC either showed a positive (iN, NH_4_^+^, TOC, oN, microbial C, microbial P) or non-significant (PO_4_^3−^, microbial N) relationship (Supplementary Data Fig. S3). For more details, see the supplementary material.

## DISCUSSION

Soil warming had limited species-specific effects on plant growth. All four species exhibited an increase in leaf N with warming, but only *Poa annua* showed a consistent positive response to warming, increasing RGR and above-ground biomass in both experiments. *Poa annua* is a ruderal and highly invasive grass. Its proliferation in disturbed environments has been attributed to high phenotypic plasticity, allowing high responsiveness to environmental changes (Chwedorzewska *et al*., 2015; Williams *et al*., 2018; Rudak *et al*., 2019). It has shown competitive nutrient acquisition in the warming Antarctic (Cavieres *et al*., 2018; Molina-Montenegro *et al*., 2019) and photosynthetic acclimation to higher temperatures in the sub-Antarctic (Ripley *et al*., 2020). *Agrostis stolonifera*, the other invasive species (Mathakutha *et al*., 2019), also showed significantly higher RGR with warming. Despite the different life-history strategies, all four of the grasses were expected to respond to nutrient release. Pulses of high nutrients occur across the island due to pinniped and seabird activity, across all habitats on which the four grasses occur (Smith, 1976, 1978). Furthermore, another study shows both *Polypogon magellanicus* and *Poa cookii* proliferate root biomass with high NO_3_^−^ supply in hydroponic culture, thus maximizing the uptake of high N pulses (Pallett *et al.*, 2024). The restricted positive response to *Poa annua* and *A. stolonifera* suggests that soil warming stimulated plant growth to a degree, but this was limited and thus only capitalized on by the invasive, ruderal species.

Warming affects plant growth both directly and indirectly. Soil warming can stimulate plant root growth and nutrient uptake (BassiriRad, 2000; Yin *et al*., 2013). In this study, while soil warming may have directly stimulated growth, the concurrent increases in soil iN and leaf N suggest that the plant responses were at least partially due to warming-induced nutrient release. Furthermore, the combined effect of fertilizer and warming did not significantly increase RGR or leaf N compared to fertilizer without warming. Only *Poa annua* showed an increase in above-ground biomass with warming between the two fertilizer treatments. This is consistent with the combined direct and indirect effects of warming having contributed to increased biomass of *Poa annua*.

Fertilizer application produced the strongest biomass response, revealing nutrient limitation to growth even under soil warming. However, fertilizer application has been used in experiments as a proxy for soil warming, implicitly assuming warming will increase nutrient release (e.g. see Jonasson *et al*., 1999; Graglia *et al*., 2001). The use of fertilizer as a proxy for soil warming is inappropriate, because nutrient release with warming may not occur or be comparably small. Furthermore, responses to warming decline with time due to microbial temperature acclimation or substrate limitation (Kirschbaum, 2004; Romero-Olivares *et al*., 2017). Fertilizer application as a warming proxy also assumes simultaneous increases in all nutrients required by plants, which may not be the case. There is evidence for a decoupled response between N and P with warming (Geng *et al*., 2017), which can affect plant biomass responses (Madan *et al*., 2007), plant nutrition and species composition (Güsewell, 2004). In this study, warming increased soil iN and not P, and microbial P per gram TOC decreased with high warming. Possible P limitations with warming that should be investigated further. This, and the evidence for soil warming having a non-significant, limited or only short-term effect on plant growth, shows that any results where fertilizer has been used as a proxy for warming need to be interpreted with caution.

The predicted increases in soil decomposition rates under warming, releasing the nutrient bottleneck to cold-ecosystem plant productivity (Chapin, 1983; Kirschbaum, 1995; Smith, 2002), should not be assumed. Many studies (across different latitudes) document significant effects of short-term warming on microbial activity and nutrient release (Hobbie, 1996; Rustad *et al*., 2001; Bai *et al*., 2013; Salazar *et al*., 2020). However, limited or non-significant responses to warming have also been reported (e.g. some sites in Rustad *et al*., 2001 and Bai *et al*., 2013), showing this is neither ubiquitous nor consistent. This study contributes to these data, showing that warming does not necessarily stimulate high nutrient release, even over short-term periods, in controlled environments, and with highly organic substrates, where the effects are expected to be strong. In the natural environment, concomitant drying with soil warming may ameliorate a warming response (Allison and Treseder, 2008; Beier *et al*., 2008); indeed, nutrient concentrations and microbial biomass increased with soil moisture in the current study. This questions the extent to which nutrient availability is limited by temperature alone in cold systems and shows the importance of testing such assumptions empirically.

The complex interplay of the mechanisms driving organic matter decomposition may effectively buffer a warming stimulus. For example, Sistla and Schimel (2013) observed seasonal decoupling of oxidative and hydrolytic enzymes, and spatial decoupling of the response size of extracellular enzymes between shallow and deep soil horizons. These separations resulted in negative feedback loops, curtailing increases in decomposer activity. Litter quality has a strong effect on decomposition and may change over time should plant nutrition or even vegetation composition change under soil warming (Rinnan *et al*., 2007; Steinauer *et al*., 2015; Ward *et al*., 2015). To this end, studies show that instead of soil warming driving changes in vegetation by influencing microbial activity and communities, changes in vegetation composition have larger, sustained influences on soil decomposition. For example, Rinnan *et al*. (2007) documented differences in microbial biomass only after 15 years of warming and accompanying changes in vegetation composition.

Changes in vegetation composition, which may be related to warming (for productivity but also phenology) and other drivers such as alien invasions, may be a more important and long-term driver of changes in soil processes. There is recent empirical evidence for vegetation change on MI, including increased overall vegetation and invasive species cover (van der Merwe *et al*., 2024). These changes have been attributed to the current warming and drying trends that the island is experiencing, and biotic invasions. Warming-induced nutrient release may have contributed to these changes, particularly for invasive species such as *Poa annua*. However, the overall small plant biomass and soil N responses to warming in this study suggest that nutrient release is probably not a major driver of current MI vegetation change.

Invasive species, including cats and mice, may represent an important driver of nutrient cycling on MI. Mice feed predominantly on soil invertebrates, limiting nutrient release through invertebrate grazing (Smith and Steenkamp, 1993; McClelland *et al*., 2018). Domestic cats, which quickly became feral following their introduction in 1949, wreaked havoc on nesting bird populations leading to the local extinction of some species, until they were eradicated (van Aarde, 1980; Bester *et al*., 2002). The decimation of bird populations may have significantly decreased allochthonous nutrient input, with ramifications for nutrient cycling and vegetation productivity (e.g. van der Merwe *et al*., 2024). Furthermore, in addition to feeding on invertebrates, the mice prey on seabirds, impacting seabird populations (Dilley *et al.*, 2018). These changes could have a large impact on plant productivity that is difficult to untangle from the effects of climate warming. The Prince Edward Islands represent a unique study site for untangling climate and invasion drivers, as the nearby Prince Edward Island (19 km from MI) has experienced the same climate trends but no invasions by cats or mice (Greve *et al*., 2017). The differences between climate and biotic drivers on current sub-Antarctic vegetation change remain to be determined. Indeed, such investigations should be given a high priority given the unique context of this island group, with the combination of simple ecological systems, high rates of climate change and substantial differences in biotic invasion histories between the two sites.

There are limitations to this study, such as the lack of a below-ground root harvest and the type of soil warming implemented. Soil warming can directly stimulate root growth (Yin *et al*., 2013), and increased N concentrations (due to increased temperature) could also stimulate root proliferation (Miller and Cramer, 2004; Hodge, 2009). The observed increase in leaf N may thus be attributable to warming-stimulated root proliferation, or the combination of this with higher soil N. Roots were not harvested in this experiment due to the difficulty of separating root biomass from the organic matter of the soil. Moreover, a preparatory trial at the same site using the same species (in hydroponics culture) showed that root responses to warming were limited. Warming had no significant influence on root biomass, and only *Poa annua* (as with the above-ground biomass in the current study) showed an increased root:shoot ratio. Regarding the type of warming implemented, this study only tested an increase in average soil temperature, but the reality of climate change is more complex. For example, there is evidence for an increase in the number of consecutive days >10 °C and consecutive dry days (Ripley *et al*., 2020; van der Merwe *et al*., 2024). The influence of these other factors, and their potential interactions with other drivers of vegetation change, needs further investigation.

## CONCLUSION

Vegetation change may have stronger influences on MI soil decomposition than increased temperature alone. Therefore, rather than altered nutrient cycling driving vegetation change, vegetation change may instead drive changes to soil nutrient cycling. On MI, there is evidence of changes in plant distributions and abundances (le Roux and McGeoch, 2008*b*; le Roux *et al*., 2013; van der Merwe *et al*., 2024), though its effect on MI soil processes is not known. This warrants further investigation, particularly given the impacts of biological invasions to the relatively simple ecological structure of sub-Antarctic islands, and the fact that warming may contribute to the spread of invasive species on these islands (Mathakutha *et al*., 2019; Ripley *et al*., 2020).

## Supplementary Material

mcaf154_Supplementary_Data
