# Supplementary material for: Warming has limited effects on plant growth through nutrient release: evidence from sub-Antarctic Marion Island
Source: Ann Bot. 2025 Jul 18;136(4):865–76. doi: 10.1093/aob/mcaf154 (PMC12464945; doi:10.1093/aob/mcaf154)
Supplement: mcaf154_Supplementary_Data [file mcaf154_supplementary_data.pdf]

Table. S1: Site details from which soil cores (n = 20) were extracted for the soil incubation experiment. Predominant vegetation, faunal influence (ranked a-e from low to high) and type is based on field observations (April 2019 – May 2020). Vertebrate presence was estimated based on ad-hoc field observations during 2019 and 2020.

Site	Vegetation complex (Smith et al. 2001)	Predominant vegetation	Vertebrate presence (2020)	Invertebrate presence
**a** Planted experiment site	Coastal salt-spray complex	Bryophyte and *Sagina procumbens* mat	None / some ad-hoc visitation by *Chionis minor* (Black-faced Sheathbills)	Earthworms present
**b** Slope complex *ca*. 400 m from the coast	Slope complex	*Austroblechum penna-marina*, some *P. cookii*	Inactive *Pterodroma macroptera* (Great-winged Petrel) nests from the previous breeding season	None
**c** Biotic slope, ≥ 3 m away from a burrowing petrel nest	Slope complex	*Austroblechum penna-marina*, some *P. cookii*	*Procellaria aequinoctialis* (White-chinned Petrel): recently active nest site	Earthworms present
**d** Biotic slope, < 1 m from a burrowing petrel nest entrance	Slope complex	*Austroblechum penna-marina*, some *P. cookii*	*Procellaria aequinoctialis* (White-chinned Petrel): recently active burrow	None
**e** Biotic site at the coast	Biotic herbfield complex	Bryophyte mat with adjacent lawn (*P. annua* and *P. magellanicus*)	High trampling and manuring by Southern Elephant Seal, Antarctic fur-seal, and some penguin species	Earthworms present

Table. S2: Soil sites where iButtons were placed *in situ* on MI from (08-11-2019 to 10-04-2020), with coordinates and mean ± SE soil temperatures (Schoombie and le Roux, *pers. comm*).

Site name	Coordinates (lat, long)	Temperature (°C)
Kampkoppie	-46.895427°, 37.611773°	5.48 ± 2.66
Mixed Pickle	-46.873683, 37.637732	5.88 ± 2.71
Repettos	-46.844362, 37.766535	6.32 ± 2.96
East Cape	-46.897869, 37.899481	6.31 ± 3.24
Kildalkey	-46.952965, 37.842466	5.47 ± 2.88

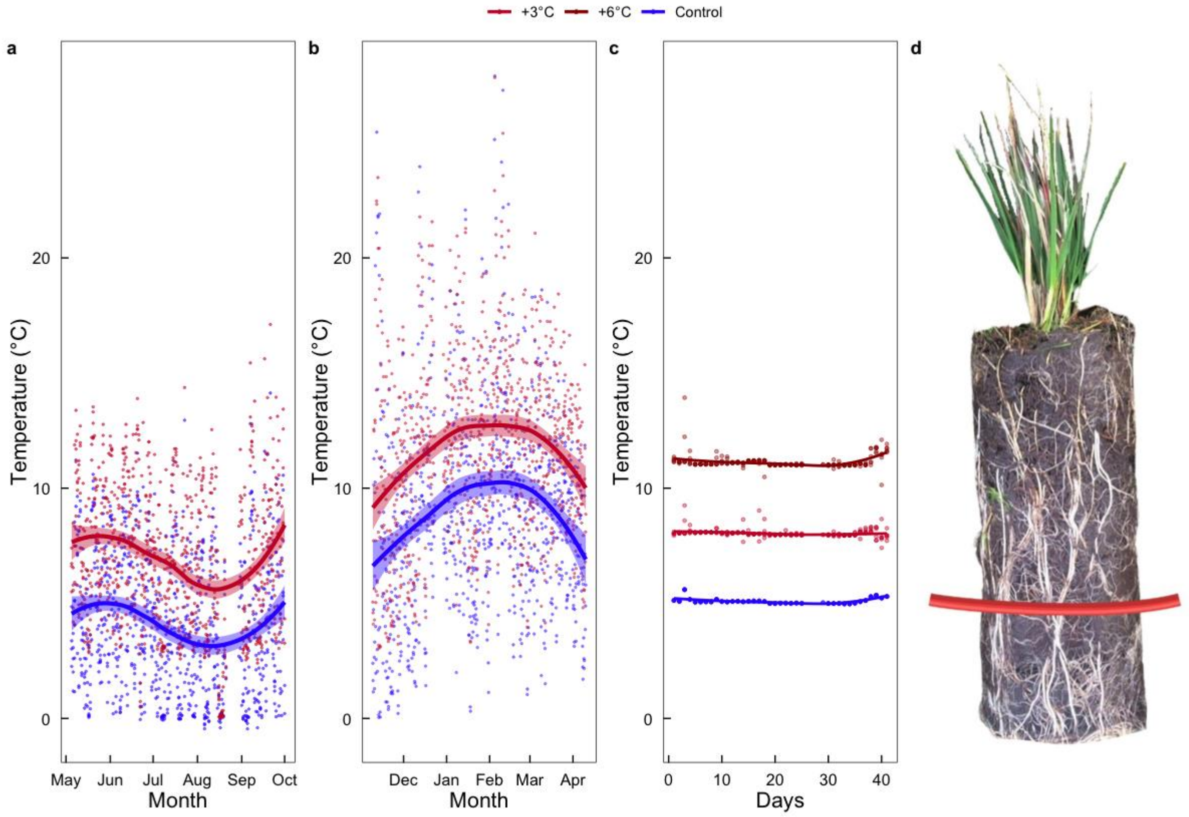

Fig. S1. Temperatures of the control (blue) and heated (red) treatments, with points showing the mean temperature per hour, and lines plotted with 'loess' model fitting: **a** in the winter MI planted experiment, **b** in the summer MI planted experiment, and **c** for the soil incubation experiment. There were two unexpected power outages, one in each planted experiment (late August and late December), resulting in no heating or data for those periods. The illustration **d** shows *P. magellanicus* in a soil core following five months of growth, with a schematic heating cable to indicate its position in the soil cores.

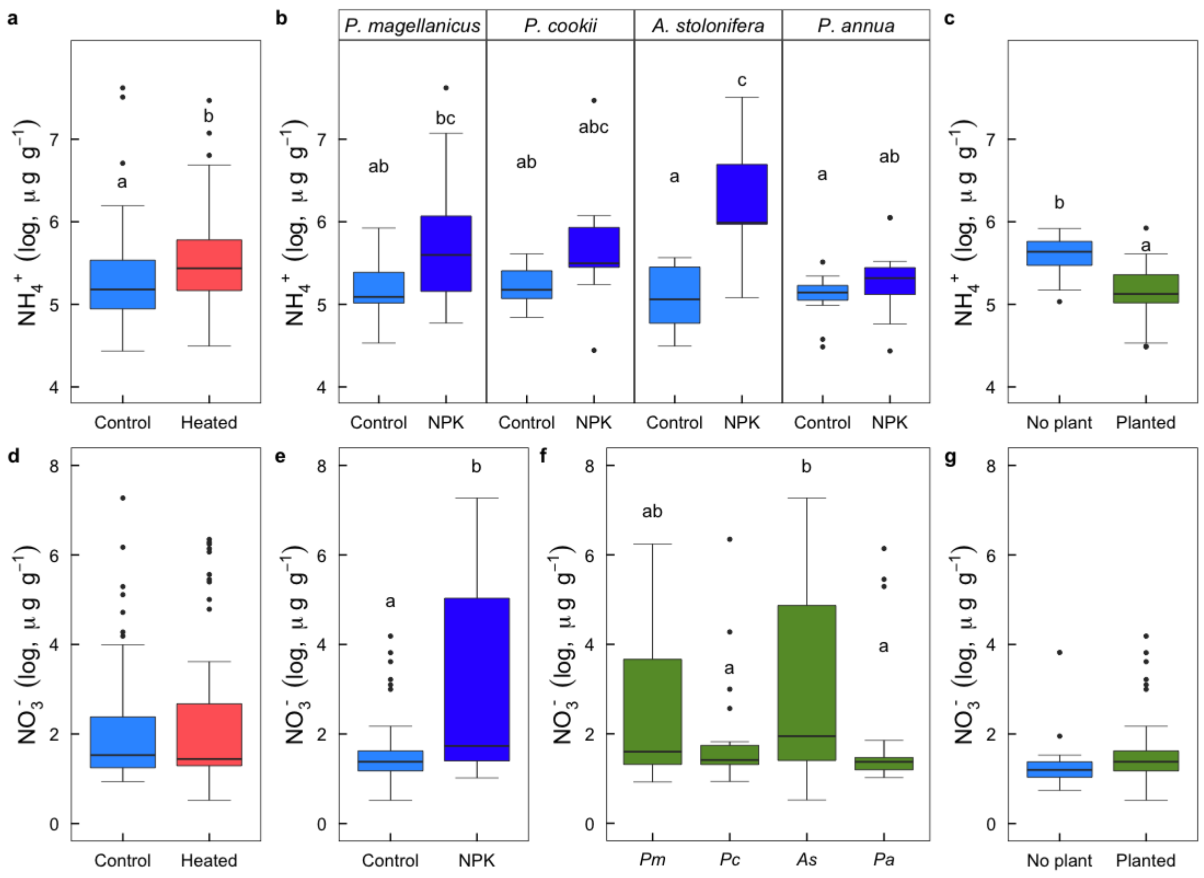

Fig. S2. Soil iN (NH_4^+ and NO_3^-) from the planted summer experiment. **a** Soil NH_4^+ concentrations between the control and heated pots, where NH_4^+ increased with heating ($F_{1,87} = 3.99$, $p = 0.049$), and **b** with NPK fertilisation, where NPK fertilisation increased soil NH_4^+ for pots with *A. stolonifera* ($F_{3,87} = 3.55$, $p = 0.018$). **c** Difference in soil NH_4^+ between planted and unplanted pots ($F_{1,57} = 18.18$, $p < 0.0001$). **d** Soil NO_3^-, where there was no significant difference in NO_3^- concentration between warming treatments. **e** NO_3^- increased significantly with NPK fertilisation ($F_{1,90} = 21.07$, $p < 0.0001$), and **f** NO_3^- was higher in pots with *A. stolonifera* than those with *P. cookii* and *P. annua* ($F_{3,87} = 3.47$, $p = 0.019$). There was no difference between soil NO_3^- in planted and unplanted pots. Significant differences between treatments and species are denoted by the absence of common letters, as determined by a Tukey HSD post-hoc test at the $\alpha = 0.05$ significance level. Data are plotted on a log scale to improve visual representation.

Soil water content

When compared between the three warming treatments, SWC showed no significant change ($F_{2,148}$ = 1.37, p = 0.26). SWC had a significant effect on the relationship between iN and warming treatment, with a negative relationship between SWC and iN in the control treatment, but more positive relationships with the warming treatments ($F_{2,173}$ = 4.01, p = 0.02; Fig. S3a). When compared to NO_3^- and NH_4^+, SWC had contrasting effects. With NO_3^-, there was an interaction between SWC and warming treatment, where all treatments resulted in a negative relationship with SWC ($F_{2,173}$ = 7.45, p = 0.0008; Fig. S3b). However, SWC regressed positively with NH_4^+ ($F_{1,12.9}$ = 6.56, p = 0.04) and this was not affected by the warming treatments (Fig. S3c). SWC and warming treatment had a significant interaction effect on soil TOC, with warming resulting in a more positive relationship between SWC and TOC ($F_{2,173}$ = 4.46, p = 0.013; Fig. S3d). Soil oN regressed positively with SWC with marginal significance ($F_{1,11}$ = 3.92, p = 0.073), with no effect of warming (Fig. S3e). There was no effect of SWC on soil PO_4^{3-} (Fig. S3f). Microbial C showed a positive relationship with SWC, and this was not affected by the warming treatment ($F_{1,124}$ = 7.55, p = 0.007; Fig. S3g). There was no evidence for a relationship between microbial N and SWC (Fig. S3h). Microbial P showed a positive relationship with SWC ($F_{1,168}$ = 21.89, p < 0.0001; Fig. S3i) and this relationship was not affected by warming.

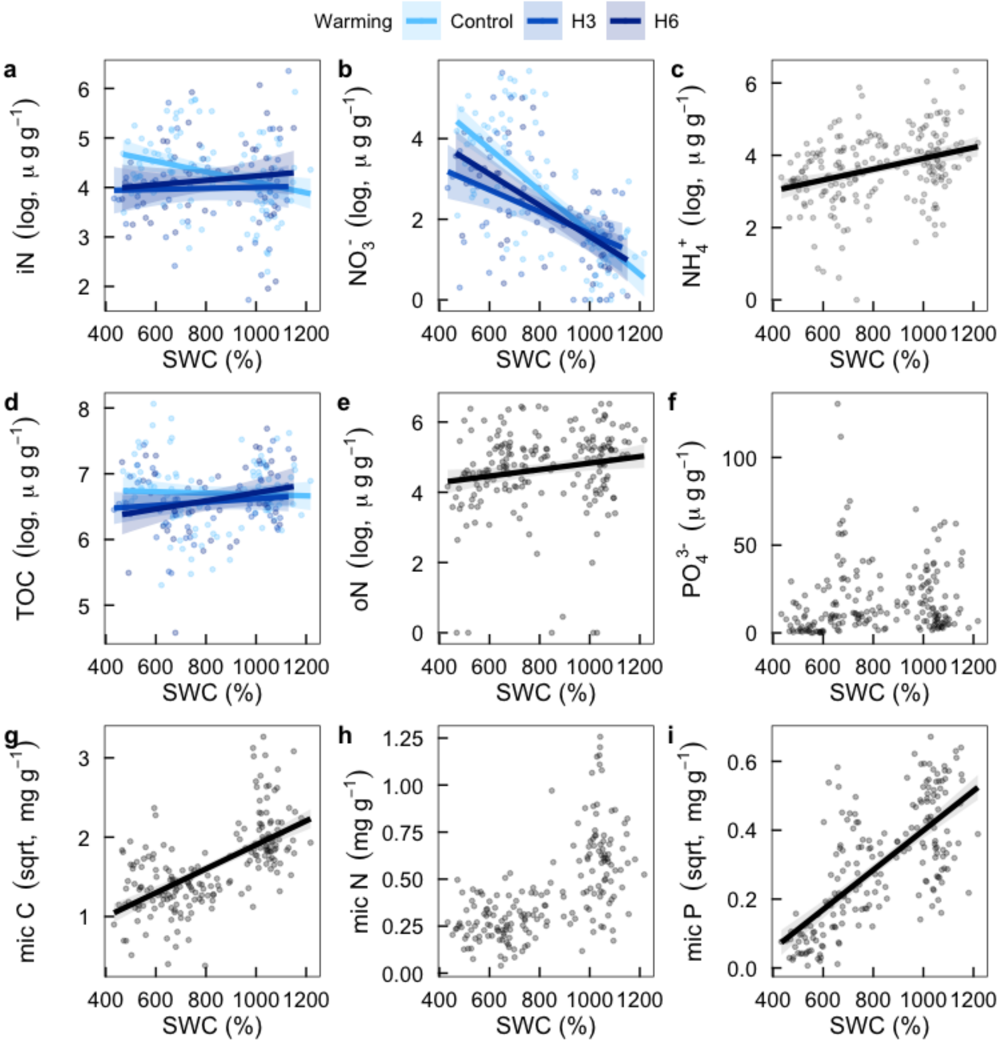

Fig. S3. Soil and microbial nutrient regressions with SWC (%). Where there was a significant effect of warming, the lines are presented in colour, but otherwise are shown in black. **a** The relationship between soil iN and SWC, which increased with warming. **b** Soil NO_3^- had a negative relationship with SWC, which was affected by the warming treatments. **c** Soil NH_4^+, **e** soil oN, **g** microbial C, and **i** microbial P had a significant positive relationship with SWC, and there was no effect of warming. **d** Soil TOC was affected by SWC and warming, and **f** soil PO_4^{3-} and **h** microbial N showed no relationship with SWC. Where data transformations were necessary to meet the assumptions of the linear models, the data are plotted with the respective transformation ($\log_e$ or square-root), and this is indicated on the y-axis.
